# Selective targeting of lectins and their macropinocytosis in urothelial tumours: translation from in vitro to ex vivo

**DOI:** 10.1007/s00418-023-02224-2

**Published:** 2023-08-03

**Authors:** Nataša Resnik, Tanja Višnjar, Tomaž Smrkolj, Mateja Erdani Kreft, Rok Romih, Daša Zupančič

**Affiliations:** 1 Institute of Cell Biology, Faculty of Medicine, University of Ljubljana, Vrazov Trg 2, 1000 Ljubljana, Slovenia; 2 Clinical Institute of Genomic Medicine, University Medical Centre Ljubljana, Ljubljana, Slovenia; 3 Department of Urology, University Medical Centre Ljubljana, Ljubljana, Slovenia; 4 Department of Surgery, Faculty of Medicine, University of Ljubljana, Ljubljana, Slovenia

**Keywords:** Endocytosis, Glycosylation, Lectins, Macropinocytosis, Urinary bladder tumours, Cancer urothelial cells

## Abstract

Urinary bladder cancer can be treated by intravesical application of therapeutic agents, but the specific targeting of cancer urothelial cells and the endocytotic pathways of the agents are not known. During carcinogenesis, the superficial urothelial cells exhibit changes in sugar residues on the apical plasma membranes. This can be exploited for selective targeting from the luminal side of the bladder. Here we show that the plant lectins Jacalin (from *Artocarpus integrifolia*), ACA (from *Amaranthus caudatus*) and DSA (from *Datura stramonium*) selectively bind to the apical plasma membrane of low- (RT4) and high-grade (T24) cancer urothelial cells in vitro and urothelial tumours ex vivo. The amount of lectin binding was significantly different between RT4 and T24 cells. Endocytosis of lectins was observed only in cancer urothelial cells and not in normal urothelial cells. Transmission electron microscopy analysis showed macropinosomes, endosome-like vesicles and multivesicular bodies filled with lectins in RT4 and T24 cells and also in cells of urothelial tumours ex vivo. Endocytosis of Jacalin and ACA in cancer cells was decreased in vitro after addition of inhibitor of macropinocytosis 5-(N-ethyl-N-isopropyl) amiloride (EIPA) and increased after stimulation of macropinocytosis with epidermal growth factor (EGF). Clathrin, caveolin and flotillin did not colocalise with lectins. These results confirm that the predominant mechanism of lectin endocytosis in cancer urothelial cells is macropinocytosis. Therefore, we propose that lectins in combination with conjugated therapeutic agents are promising tools for improved intravesical therapy by targeting cancer cells.

**Supplementary Information:**

The online version contains supplementary material available at 10.1007/s00418-023-02224-2.

## Introduction

The urothelium is the stratified epithelium of the urinary bladder and is the main origin of cancer transformation leading to bladder cancer (Shin et al. 2014). In the healthy bladder, the highly differentiated superficial umbrella cells express large amounts of four uroplakins (UPIa, UPIb, UPII and UPIIIa) that form urothelial plaques in the apical plasma membrane (Yu et al. 1994; Wu et al. 1994; Wu et al. 1990; Hudoklin et al. 2011; Kachar et al. 1999; Porter et al. 1967). UPII is not glycosylated, whereas UPIa, UPIb and UPIIIa are all glycosylated with N-linked glycans (Yu et al. 1994; Wu and Sun 1993). UPIa and UPIb are glycosylated by mannose-rich structures and complex glycans, respectively (Xie et al. 2006). UPIIIa contains complex glycans capped by sialic acid in α(2,3)- and α(2,6)-linkage with galactose (Gal) and by the Gal-α(1,3)-Gal (Malagolini et al. 2000). UP expression and glycosylation profiles are altered during urothelial preneoplastic changes and early urothelial carcinogenesis and are downregulated at later stages of urothelial carcinogenesis (Zupancic 2013; Zupancic et al. 2020; Zupancic et al. 2011a; Ogawa et al. 1999; Wu et al. 1998; Zupančič et al. 2011b; Kątnik-Prastowska et al. 2014). It is not known how these particular glycans are altered during bladder carcinogenesis, but it seems that the proteoglycans, glycoproteins and glycosphingolipids, together with their ligands, play a crucial role in the malignant transformation, invasion and metastasis of bladder cancer (Ohyama 2008) and could be exploited for targeted drug delivery by endocytosis into cancer urothelial cells.

Bladder cancer is divided into non-muscle invasive (NMIBC) and muscle invasive bladder cancer (MIBC). NMIBC tumours are confined to the urothelium (low-grade papillary carcinoma called papilloma and stage Ta) or invade the lamina propria below the urothelium (stage T1). MIBCs invade the muscle (stage T2) or perivesical adipose tissue and sometimes adjacent organs (stage T3 or T4, respectively). Useful in vitro models for NMIBC (stage Ta) are the low-grade human urothelial papilloma cell line RT4 and for MIBC (stage T2) the high-grade urothelial cancer cell line T24. Despite successful removal of NMIBC tumours by transurethral resection of the bladder (TURB) and intravesical instillation of mitomycin C or Bacillus Calmette Guerin (BCG), the recurrence rate is high (50–70%) (Ritch et al. 2020). Approximately 20% of recurrences progress to MIBC, which has a high risk of metastasis with poor prognosis (Aragon-Ching et al. 2018). Despite numerous novel experimental approaches, there has been little progress in developing more selective therapies that could reduce local or systemic side effects. Therefore, more targeted delivery of cytotoxic drugs to cancer cells is beneficial to avoid drug toxicity and improve drug efficacy. In the case of bladder cancers, of which > 75% are NMIBC at initial diagnosis (Kamat et al. 2016), it is possible to use the intravesical route for targeted delivery of site-specific drugs that could be administered through a catheter to avoid systemic use.

Intensive research is being conducted on various strategies for targeted drug delivery to urothelial tumours (Zacchè et al. 2015; Yoon et al. 2020; Heath and Rosenberg 2021). Among them, plant lectins such as WGA (wheat germ agglutinin from *Triticum vulgaris*), Jacalin (from *Artocarpus integrifolia*), ACA (from *Amaranthus caudatus* agglutinin) and DSA (from *Datura stramonium* agglutinin) are being investigated and proposed as a promising approach for selective drug delivery to bladder cancer cells due to changes in sugar residues on the cell surface (Neutsch et al. 2014, 2012, 2011, 2013; Plattner et al. 2008; Zupancic et al. 2014; Kreft et al. 2009b; Azevedo et al. 2017). Jacalin and ACA show high affinity for T-antigen (Core1) and Tn-antigen (alpha-N-acetylgalactosamine)-bound peptides and Jacalin also for Core3 and sialyl-T (ST)-bound peptides (Tachibana et al. 2006). DSA recognises galactosyl (β-1,4) N-acetylglucosamine oligomers and prefers chitobiose or chitotriose over a single *N*-acetylglucosamine residue. We have previously demonstrated in the rodent bladder cancer model that Jacalin, ACA and DSA show specific targeting of transformed cells. Jacalin and DSA discriminated between normal and cancer urothelial cells, while ACA showed differential binding during cancer progression (Zupancic et al. 2014).

It is known that cancer cells from different types of tumours engulf large amounts of extracellular fluid, particulate cargo and membranes with specific sugar residues (Xiao et al. 2021). We have shown that poorly differentiated urothelial cells have increased endocytotic activity compared to highly differentiated superficial urothelial cells (Kreft et al. 2009b; Tratnjek et al. 2017; Lojk et al. 2018). Since cancer urothelial cells and superficial cells of tumours do not reach terminal differentiation, it is important to understand the mechanism of lectin endocytosis to use lectins as targeting ligands for a drug delivery system.

To investigate the selective targeting and endocytosis mechanism of lectins, the lectins Jacalin, ACA and DSA with different carbohydrate specificities were selected. To increase the significance of our results and bring them closer to a potential clinical application in urology, we combined experiments on cancer urothelial cells in vitro with urothelial tumours ex vivo. Our results show that lectins can be used for selective targeting of urothelial tumours and that the main mechanism of lectin endocytosis in cancer urothelial cells is macropinocytosis.

## Materials and methods

### Materials

FITC-conjugated lectins from *A. integrifolia* (Jacalin; FL-1151) and *D. stramonium* (DSA; FL-1181) were purchased from Vector Laboratories (Burlingame, CA, USA) and FITC-conjugated lectins from *A. caudatus* (ACA; 21,510,290) were purchased from GlycoMatrix (Dublin, OH, USA). Colloidal gold-conjugated lectins Jacalin-15 nm gold (GP-6301–15), ACA-10 nm gold (GP-8201–10) and DSA-5 nm gold (GP-5701–5) were purchased from EY Laboratories (San Mateo, CA, USA). Lectin inhibitory molecule bovine submaxillary mucin for ACA, galactose for jacalin and chitin hydrolysate for DSA were also purchased from Vector Laboratories. Rabbit polyclonal anti-uroplakin (UP) primary antibody was a kind gift from Prof. Dr. Tung-Tien Sun, Department of Cell Biology, New York University Medical School (Wu et al. 1990). The following rabbit polyclonal antibodies were also used: anti-flotillin (ab41927, Abcam), anti-clathrin (610,499, BD Transductions), anti-caveolin (ab18199, Abcam) and anti-dynamin 2 (ab65556, Abcam). Goat anti-rabbit IgG AlexaFluor 488 (A11008, Invitrogen) and goat anti-rabbit IgG AlexaFluor 555 (A21428, Invitrogen) secondary antibodies were used. Vectashield mounting medium with DAPI, a nuclei marker, was obtained from Vector Laboratories.

### Cell cultures for in vitro experiments

Human bladder cancer cell lines RT4 and T24 (ATTC, Manassas, VA) were grown in Advanced Dulbecco's Modified Eagle’s Medium (A-DMEM)/F12 medium (1:1) supplemented with 5% FBS, 10,000 U/ml penicillin and 10,000 µg/ml streptomycin (Gibco, Invitrogen, Vienna, Austria) at 37 °C in a humidified atmosphere containing 5% CO_2_. Experiments were performed after growing RT4 and T24 cells (5 × 10^4^ cells/cm^2^) for 1 week. The use of porcine urinary bladders for preparation of primary urothelial cells was approved by the Veterinary Administration of the Slovenian Ministry of Agriculture and Forestry in compliance with the Animal Health Protection Act and the Instructions for Granting Permits for Animal Experimentation for Scientific Purposes. Normal porcine urinary bladders were obtained from a local abattoir and primary and secondary cultures of normal porcine urothelial cells (NPU) were prepared in our laboratory as described in detail previously (Višnjar et al. 2017; Visnjar et al. 2012; Visnjar and Kreft 2013). Briefly, urothelial cells were scraped from the bladder wall and collected in UroM medium containing MCDB153/A-DMEM (1:1), 2.5% FBS, 0.1 mM phosphoethanolamine, 0.5 µg/ml hydrocortisone, 5 µg/ml insulin, 4 mM glutamax, 10,000 U/ml penicillin and 10,000 µg/ml streptomycin. Primary and subsequent secondary cultures (2 × 10^5^ cells/cm^2^) were grown to confluence in UroM medium containing 0.9 mM calcium and 2.5% FBS (Gibco) and then transferred to UroM medium containing 2.7 mM calcium and no FBS for 3 weeks to achieve urothelial differentiation. These secondary cultures were used for immunolabelling and in vitro lectin binding assays as well as endocytosis analyses. Culture media and supplements were purchased from Sigma (Taufkirchen, Germany) unless otherwise stated.

### Patients and sampling for ex vivo experiments

The study was conducted in accordance with the 1964 Helsinki Declaration and its later amendments and approved by the National Medical Ethics Committee of Slovenia, No. 101/11/14. The study population consisted of 10 patients with bladder cancer who underwent TURB in the Department of Urology, University Medical Centre Ljubljana, Slovenia. Informed consent was obtained from all patients prior to tissue retrieval.

For transmission electron microscopy (TEM), immuno-electron microscopy and lectin analysis of endocytosis ex vivo, a sample of the urothelial tumour was obtained from each patient by cold cup biopsies. For immunolabelling and lectin binding assays, normal urothelium was also taken cystoscopically 2 cm away from the tumour. The biopsies captured the urothelium and lamina propria. For pathological staging and grading of all samples, the pathologist (from the Institute of Pathology, Faculty of Medicine, University of Ljubljana) used the AJCC cancer staging manual (Amin et al. 2017). Urothelial cancers were diagnosed as papilloma (1 sample), non-invasive low-grade urothelial carcinoma-Ta, l. g. (3 samples), invasive low-grade urothelial carcinoma with invasion into the lamina propria-T1, l. g. (2 samples), invasive urothelial carcinoma high-grade with invasion into the lamina propria-T1, h. g. (2 samples) and invasive urothelial carcinoma high-grade with invasion into the muscularis propria-T2, h. g. (2 samples). Of ten cystoscopically normal urothelial specimens, six were classified as normal because histopathological examination revealed that they showed no signs of hyperplasia or dysplasia.

### Lectin binding assay

#### In vitro

To analyse lectin binding only, NPU, RT4 and T24 cells were cooled to 4 °C for non-specific inhibition of endocytosis. This step ensured lectin binding without internalisation. Cells were washed with ice-cold PBS and incubated with FITC-conjugated lectins (20 μg/ml) for 30 min at 4 °C in the dark. For control, lectin binding with inhibitory molecules was performed. Prior to the incubation of cells with FITC-conjugated lectins, each lectin (20 μg/ml) was incubated with inhibitory molecule for 1 h at room temperature in the dark. A concentration of 0.75 M bovine submaxillary mucin for ACA and 1.11 M galactose for Jacalin as well as a stock solution of chitin hydrolysate for DSA was used. Cells were then incubated with conjugates of lectin and inhibitory molecules for 30 min at 4 °C in the dark. Afterwards, cells were washed three times with PBS and fluorescence was measured in live cells to determine the amount of lectin binding. Cells were fixed with 4% formaldehyde for 15 min at 4 °C to perform fluorescence microscopy and immunolabelling of UPs. Fluorescence intensity of FITC was measured in live cells with a microplate reader (Safire^2^, Tecan, Mannedorf, Switzerland) to determine the amount of lectin binding. Binding of lectins to the apical plasma membrane was imaged with a fluorescence microscope (AxioImager Z.1, Zeiss, Germany) using an oil immersion objective (63 × oil/NA 1.40) and optical sectioning was performed with ApoTome (Zeiss). The amount of lectin binding to the cells was presented as the average of the absolute fluorescence intensity (a.u.) in each culture. The measurements were performed at least in three independent experiments, each experiment in triplicate.

#### Ex vivo

Biopsy samples were incubated with FITC-conjugated lectins (20 μg/ml) in UroM medium. After incubation in the dark for 30 min at 4 °C, the samples were washed with PBS. Samples were then fixed in 4% formaldehyde for 30 min at 4 °C, washed with PBS, placed in embedding medium (OCT, Tissue Tek, The Netherlands) and frozen in liquid nitrogen. The frozen tissue blocks were cut perpendicular to the luminal surface of the urothelium; 7 μm serial sections were made and air-dried for 2 h at room temperature.

To visualise lectin binding, the first cryosection of each sample was mounted in Vectashield with DAPI and imaged with a fluorescence microscope (Eclipse TE300, Nikon, Japan). Consecutive cryosections from each biopsy sample were used for immunolabelling.

### Immunolabelling of uroplakins

#### In vitro

NPU, RT4 and T24 cells were fixed with 4% formaldehyde for 15 min at 4 °C and then incubated with 0.5% BSA, 0.1% saponin, 0.1% gelatin, 50 mM NH_4_Cl and 0.02% NaN_3_ for 30 min to block non-specific labelling. Then, the cells were incubated with anti-UP primary antibody (1:1000). Cells were then washed with PBS and incubated with AlexaFluor 555-conjugated goat anti-rabbit IgG secondary antibody (1:400) for 1 h at 37 °C. Cells were mounted in Vectashield with DAPI and imaged with a fluorescence microscope (AxioImager Z.1).

#### Ex vivo

Cryosections obtained after the ex vivo lectin binding assay were washed with PBS and pre-incubated with 5% fetal calf serum (FCS) and 1% BSA in PBS for 1 h at 37 °C. Then, the sections were incubated with the anti-UP primary antibody (1:1000) in 1% BSA in PBS at 4 °C overnight. For the negative controls, incubation with the primary antibody was omitted or the specific antibody was replaced with a non-relevant antibody. After washing, sections were incubated with AlexaFluor 555-conjugated goat anti-rabbit IgG secondary antibody (1:400) for 1 h at room temperature, washed with PBS and mounted in Vectashield with DAPI. Cryosections were examined with a fluorescence microscope (Eclipse TE300).

### Immuno-electron microscopy of uroplakins

Biopsies were cut into 1-mm^3^ blocks and fixed in 2% formaldehyde and 0.05% glutaraldehyde for 1 h at room temperature. Samples were dehydrated in 30% ethanol at 0 °C, 55% ethanol at −15 °C, 70% ethanol at −30 °C and 100% ethanol at −50 °C and gradually infiltrated in 100% Lowicryl HM20 (Polysciences, Germany). Samples were polymerized under UV light. Ultrathin sections were cut and collected on gold grids. Nonspecific labelling was blocked with PBS buffer containing 0.1% fish gelatine, 0.8% BSA and 5% FCS. Then, UPs were immunolocalized by anti-UPs primary antibody (1:5000). After washing with PBS, UPs were detected with anti-rabbit IgG secondary antibodies conjugated to 5 nm or 10 nm gold (Jackson ImmunoResearch, Cambridgeshire, United Kingdom). Sections were washed with PBS followed by distilled water. Five nm gold was silver-enhanced for 4 min with IntenSE (Amersham, UK). Ultrathin sections were counterstained with uranyl acetate and lead citrate (Merck, Darmstadt, Germany). For negative controls the protocols were the same, except omitting the primary antibody or incubating the sections with rabbit serum. Sections were analysed with transmission electron microscope (Philips CM100, The Netherlands) at 80 kV and micrographs were taken with an AMT camera (Advanced Microscopy Techniques Corp., Woburn, MA, USA).

### Western blotting

NPU, RT4 and T24 cells were lysed in RIPA buffer (EMD Millipore, Darmstadt, Germany) complemented with protease and phosphatase inhibitors (100 × Halt Cocktail, Thermo Scientific, 78,441). Protein concentration was determined with BCA Protein Assay Kit (Thermo Scientific); 5 µg protein per lane was loaded and size-fractionated on the 4–20% Novex Tris–glycine gels (Thermo Scientific) and transferred to Hybond ECL nitrocellulose membranes (Amersham Biosciences, Buckinghamshire, UK). Membranes were blocked in 5% non-fat dry milk in PBS containing 0.1% Tween-20 (Sigma) for 1 h at room temperature and then incubated overnight at 4 °C with the anti-UP primary antibodies (1:5000; antibodies bind strongly to UPIIIa-47 kDa and weakly to UPIa-27 kDa, UPIb-28 kDa and UPII-15 kDa) and with the anti-rabbit IgG secondary antibodies conjugated to HRP (Sigma, 1:1000) for 2 h at room temperature. Blots were stripped with Restore Western Blot Stripping Buffer (Pierce, Rockford, IL) and incubated with mouse monoclonal anti-β-actin primary antibodies (A2228, 1:2000, Sigma) to confirm equal protein loading. Anti-mouse IgG secondary antibodies conjugated to HRP were used (Sigma, 1:1000) for 2 h at room temperature. Membranes were finally probed with chemiluminescent substrate (SuperSignal West Pico Plus, Thermo Fisher Scientific). Chemiluminescence was detected with iBright FL1500 imaging system (Thermo Fisher Scientific).

### Endocytosis analysis

#### Immunolabelling in vitro

To study flotillin-, caveolin- and clathrin-mediated endocytosis, the NPU, RT4 and T24 cells were incubated with FITC-conjugated lectins for 1 h at 37 °C. Clathrin-independent/dynamin-dependent endocytosis was examined in RT4 and T24 cells. Cells were washed with PBS and fixed in 4% formaldehyde in PBS for 15 min at 4 °C. After blocking with 0.5% BSA, 0.1% saponin, 0.1% gelatin, 50 mM NH_4_Cl and 0.02% NaN_3_ for 30 min, the cells were incubated with rabbit polyclonal anti-flotillin antibody (1:200), rabbit polyclonal anti-caveolin antibody (1:200), mouse monoclonal anti-clathrin antibody (1:200) and rabbit polyclonal anti-dynamin 2 antibody (1:200) for 1 h at 37 °C. Cells were then washed with PBS and incubated with AlexaFluor555-conjugated anti-rabbit IgG or anti-mouse IgG secondary antibodies (1:400) for 30 min at 37 °C. Cells were washed and mounted in Vectashield-DAPI and imaged using a fluorescence microscope (AxioImager Z.1) and optical sections were taken (Apotom, Zeiss).

#### Endocytosis assays in vitro

To analyse macropinocytosis of lectins, RT4 and T24 cells were incubated with each FITC-conjugated lectin and 0.5 mg/ml TRITC-conjugated low-molecular-weight dextran (3 kDa, Thermo Fisher Scientific, Waltham, MA, USA) or 0.5 mg/ml TRITC-conjugated high molecular weight dextran (70 kDa, Thermo Fisher Scientific, Waltham, MA, USA) for 1 h at 37 °C. Both dextrans, 3 kDa and 70 kDa, are capable of labelling macropinosomes (Chen et al. 2018); 70 kDa dextran enters cells predominantly via clathrin- and dynamin-independent and amiloride-sensitive macropinocytosis (Li et al. 2015), making it an established marker for macropinocytosis (Commisso et al. 2013). Cells were fixed with 4% formaldehyde for 15 min at 4 °C, washed and embedded in Vectashield-DAPI. Imaging was performed using a fluorescence microscope (AxioImager Z.1).

To inhibit macropinocytosis 50 µM EIPA [5-(N-ethyl-N-isopropyl)amirolide, A3085, Sigma] or to inhibit clathrin-mediated endocytosis 100 µM Dyngo (ab120689, Abcam) was added to T24 and RT4 cells for 1 h at 37 °C. Cells were then incubated with FITC-conjugated lectins or TRITC-conjugated dextran (3 kDa) together with EIPA or Dyngo for a further 1 h at 37 °C. The total incubation time for the inhibitors was 2 h. The same protocol was used to stimulate macropinocytosis with 500 nM EGF. Control cells were left untreated with EIPA, Dyngo, dextran or EGF and incubated with FITC-conjugated lectins only. After incubation, cells were washed and the intensity of FITC-conjugated lectin or TRITC-conjugated dextran fluorescence was measured using a microplate reader (Safire^2^, Tecan, Mannedorf, Switzerland). The number of lectins in the cells was expressed as the ratio between the average fluorescence intensity after treatment and an average fluorescence intensity of control cells. Cells that were incubated with TRITC-conjugated dextran (70 kDa) were fixed with 4% formaldehyde for 15 min at 4 °C and labelled with 16.7 μg/ml FITC-conjugated phalloidin (Sigma, Germany) in PBS for 30 min at room temperature. Samples were embedded in Vectashield with DAPI. Imaging was performed using a fluorescence microscope (AxioImager Z.1).

#### Endocytosis analysis with transmission electron microscopy in vitro and ex vivo

Biopsy samples and RT4 and T24 cells were incubated with gold-conjugated lectins (1 μg/ml) for 1 h at 37 °C. Biopsies and cells were washed with PBS and fixed with 4% formaldehyde and 2.5% glutaraldehyde in 0.1 M cacodylate buffer for 2.5 h at 4 °C. They were then washed in 0.33 M sucrose in cacodylate buffer and post-fixed with 1% OsO_4_ for 1 h at room temperature. They were then dehydrated and embedded in Epon (Serva, Heidelberg, Germany). The ultrathin sections were stained with uranyl acetate and lead citrate. Samples were analysed with transmission electron microscope (Philips CM100 at 80 kV) and micrographs were taken with an AMT camera (Advanced Microscopy Techniques Corp., Woburn, MA, USA).

### Statistics

Statistical analyses were performed using Microsoft Excel and Graph Pad Prism version 5 (Graph Pad Software Inc., La Jolla, CA). A two-way analysis of variance ANOVA (Bonferroni's post hoc test) was performed to test whether differences between groups were significant. Statistical significance was defined as *p < 0.05 or **p < 0.001. All data were expressed as mean ± standard error, and experiments were performed in at least three independent experiments, each experiment in triplicate.

## Results

### Decreased expression of uroplakins is similar in in vitro and ex vivo models of bladder cancer

**Fig. 1:**
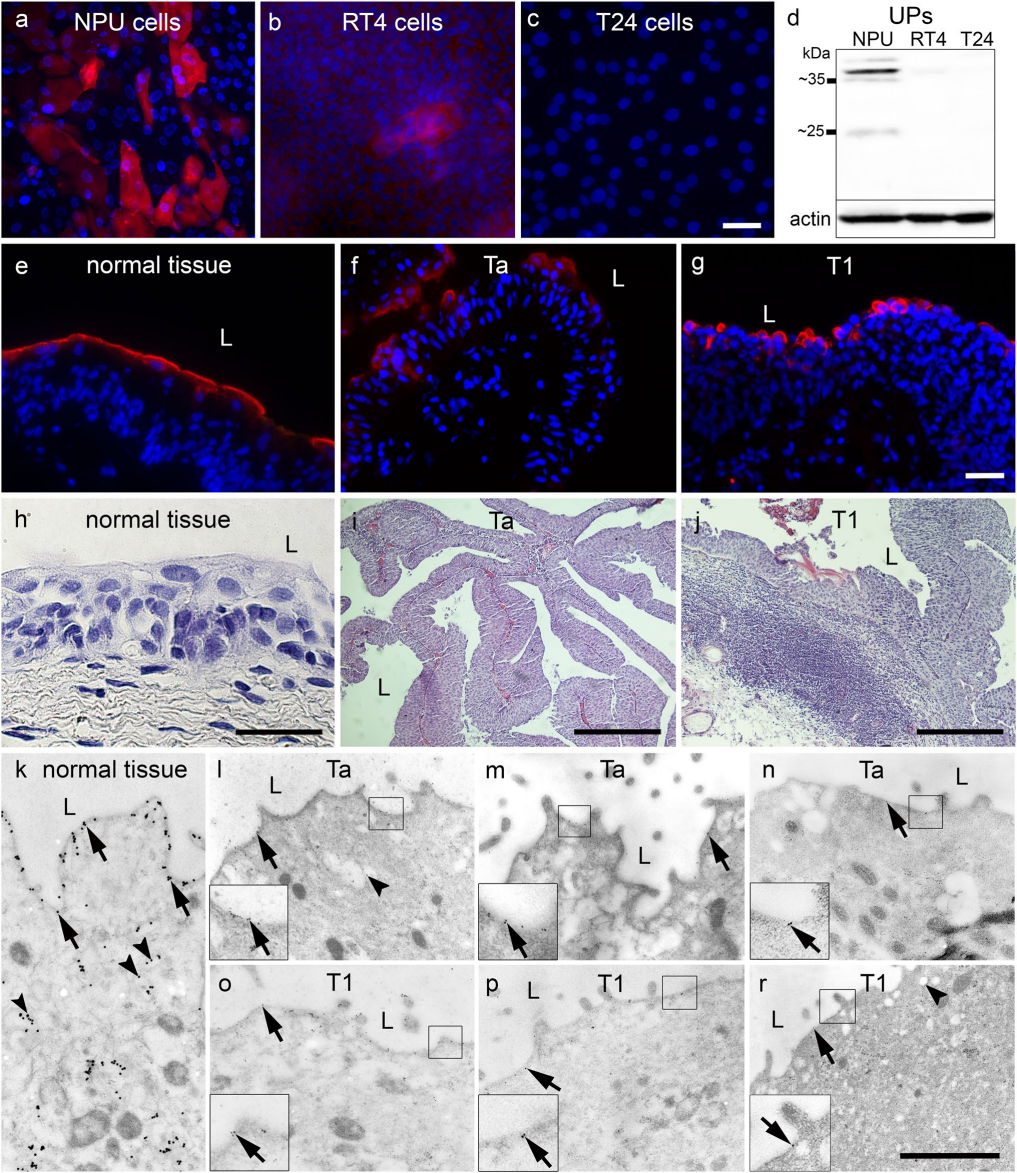
UP expression in urothelial normal and cancer cells in in vitro and ex vivo models. UP expression (red) is highest in NPU cells (**a**) and in normal tissue (**e**, **k**). UP expression is restricted to individual RT4 cells (**b**) and to individual superficial cells of Ta (**f**, **l**–**n**) and T1 tissue samples (**g**, **o**–**r**). T24 cells do not express UPs (**c**). Immunoblotting of UPs (upper strong band corresponds to UPIIIa-47 kDa and weak lower band corresponds to UPIa-27 kDa) shows strong expression in NPU cells, very weak expression in RT4 cells and no expression in T24 cells (**d**). Staining with haematoxylin and eosin shows that the morphology of the urothelium is altered in Ta (**i**) and T1 (**j**) tumours compared to normal urothelium (**h**). Immuno-electron microscopy shows abundant UPs in the apical plasma membrane (arrows) and in discoidal vesicles (arrowheads) in superficial cells of normal urothelium (**k**). In the superficial cells of Ta (**l**–**n**) and T1 (**o**–**r**) tumours, immunolabelling of UPs in the apical plasma membrane (arrows) and in round vesicles (arrowheads) is weaker. The insets in the left corners (**l**–**r**) are 250% magnifications of marked region. L = lumen. Scale bar: **a**–**h** 50 µm, **i**–**j** 100 µm, **k**–**r** 1 µm

UPs are differentiation-dependent and urothelial-specific transmembrane proteins that form urothelial plaques (Hu et al. 2005; Sun et al. 1999). Their expression has been shown to decrease during urothelial carcinogenesis (Huang et al. 2007; Ogawa et al. 1999; Zupančič et al. 2011a). Here, we examined the differentiation state of urothelial cells in in vitro and ex vivo models using UP immunolabelling (Fig. 1). The strongest reaction to UPs was observed in NPU cells (Fig. 1a), while only individual RT4 cells were positive for UPs (Fig. 1b) and T24 cells were negative (Fig. 1c) for UPs. Immunoblotting also confirmed the expression of UPs in NPU cells, minimal expression of UPs in RT4 and no expression of UPs in T24 cells (Fig. 1d).

Ex vivo, the morphology of the urothelium in Ta and T1 tumours was altered in comparison to normal urothelium (Fig. 1h–j). Normal urothelial cells showed UP expression in the apical plasma membrane (Fig. 1e, k). The urothelium of bladder cancers showed reduced expression of UPs regardless of stage Ta or T1 (Fig. 1f, g, l–r). In addition, immuno-electron microscopy showed that the superficial urothelial cells of the normal urothelium contained UPs in the apical plasma membrane and in discoidal vesicles (Fig. 1k), whereas in stages Ta and T1 there were fewer UPs than in the normal tissue in the apical plasma membrane and only a few UPs in round vesicles (Fig. 1l–r).

The results of immunolabelling of UPs confirmed that NPU cells reached a highly differentiated state comparable to normal human urothelium. However, the expression of UPs was decreased in cancer urothelial cells in vitro and ex vivo. Thus, we conclude that NPU cells correspond to normal urothelium, whereas RT4 and T24 cells correspond to Ta and T1 tumours, respectively, and do not reach a high differentiation state. These results are consistent with our previously published data (Zupancic et al. 2014; Tratnjek et al. 2017; Kreft et al. 2009a; Visnjar et al. 2017; Jerman et al. 2021; Višnjar et al. 2017).

### Lectins bind selectively to cancer urothelial cells in ex vivo and in vitro models

**Fig. 2:**
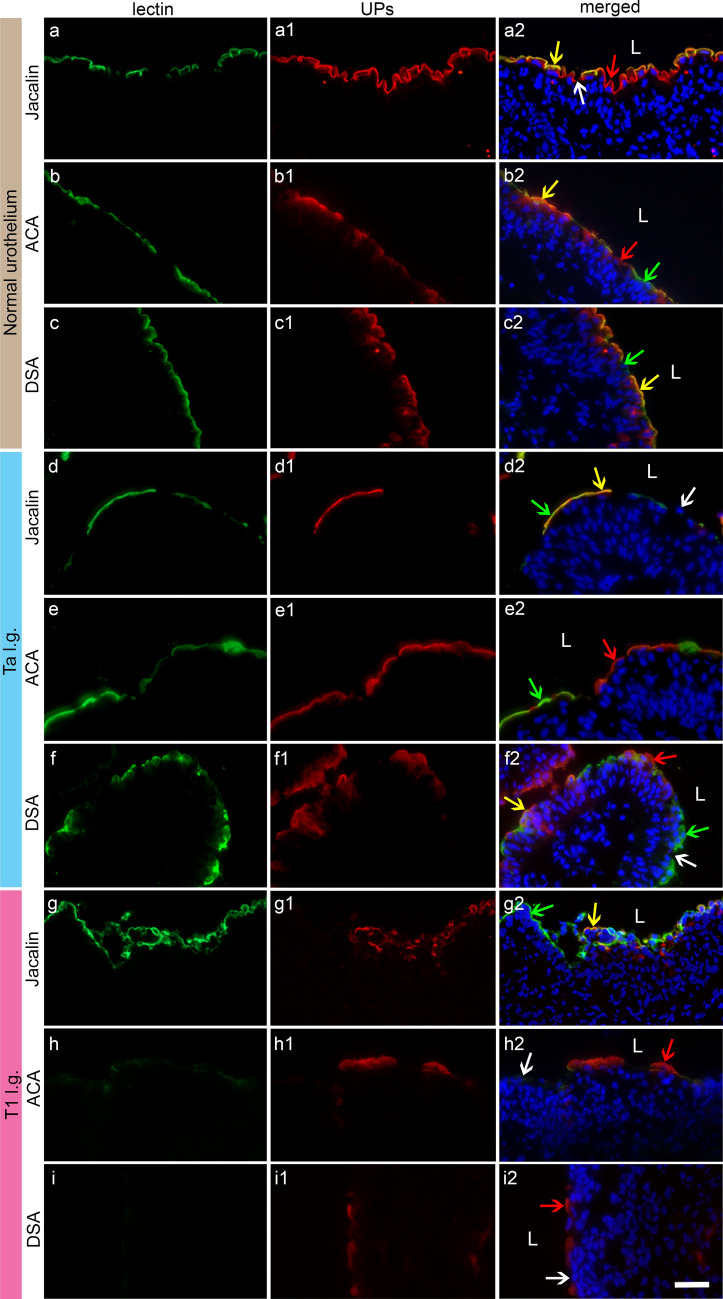
Lectins bind selectively to superficial urothelial cells in normal and cancer biopsy samples ex vivo. The apical plasma membrane of normal urothelial cells (**a**–**c2**) and cells in Ta tumours (**d**–**f2**) is labelled with Jacalin, ACA and DSA (green). The apical plasma membrane of urothelial cells in T1 tumours (**g**–**i2**) is labelled with Jacalin (**g**) and is negative for ACA (**h**) and DSA (**i**). Differentiated superficial urothelial cells in normal urothelium (**a1**, **b1**, **c1**), Ta l.g. (**d1**, **e1**, **f1**) and T1 l.g. (**g1**, **h1**, **i1**) express UPs (red). In merged images colocalisation or no colocalisation between lectins and UPs is clearly observed: UP-positive cells with lectin labelling = colocalisation (**a2**, **b2**, **c2**, **d2**, **f2**, **g2**; yellow arrows); UP-negative cells without lectin labelling = no colocalisation (**a2**, **d2**, **f2**, **h2**, **i2**; white arrows); UP-positive cells without lectin labelling = no colocalisation (**a2**, **b2**, **e2**, **f2**, **h2**, **i2**; red arrows); UP-negative cells with lectin labelling = no colocalisation (**b2**, **c2**, **d2**, **e2**, **f2**, **g2**; green arrows). L = lumen; l.g. = low grade. Scale bar for all images: 50 µm

**Fig. 3:**
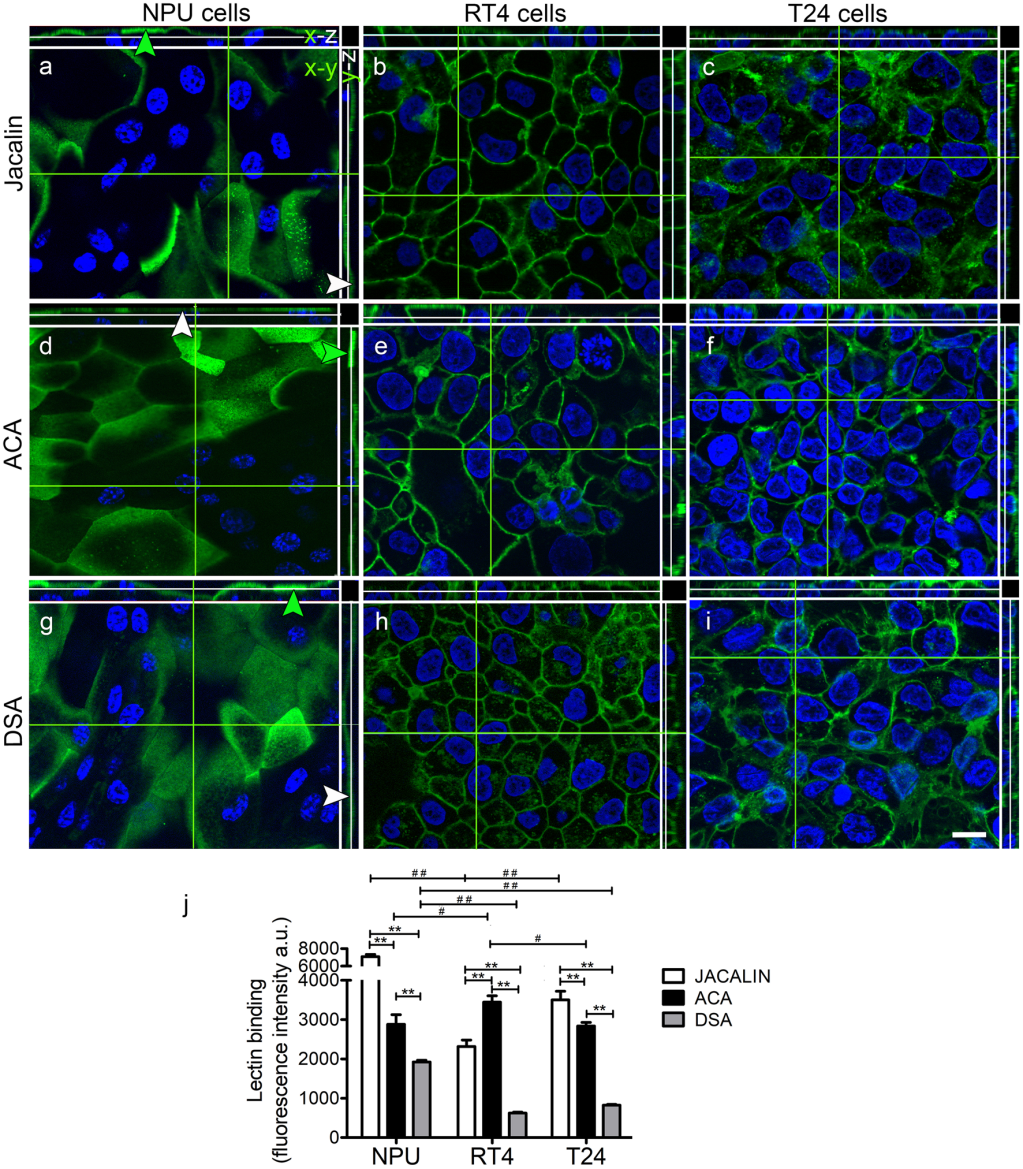
Selective lectin binding to urothelial normal and cancer cells in vitro. FITC-conjugated lectins Jacalin (**a**, **b**, **c**), ACA (**d**, **e**, **f**) and DSA (**g**, **h**, **i**) (green) bind to the plasma membrane of NPU, RT4 and T24 cells. The plasma membranes of all T24 and RT4 cells are uniformly labelled with Jacalin, ACA and DSA, whereas NPU cells have stronger (green arrowheads) and weaker (white arrowheads) labelling with Jacalin, ACA and DSA. The green lines on the x–y sections correspond to the white lines on the x–z and y–z views of the optical sections. NPU cells have a heterogeneous morphology shown on x–y sections of the apical parts of the cells. (**j**) Lectin binding to NPU, RT4 and T24 cells is calculated from absolute values of FITC fluorescence intensities. Shown are average values ± SE; *p < 0.05, **p < 0.001. Scale bar: 10 µm

We have previously shown that Jacalin and ACA exhibit selective binding to cancer and normal urothelium in rats and mice (Zupancic et al. 2014). Here, human biopsy samples from normal urothelium and bladder cancer (Ta and T1) were used to assess lectin binding ex vivo (Fig. 2) and compare with lectin binding to NPU, RT4 and T24 cells in vitro (Fig. 3). We analysed the correlation between the binding of Jacalin, ACA and DSA to urothelial cells and UP labelling corresponding to their differentiation state.

In ex vivo samples, Jacalin (Fig. 2a), ACA (Fig. 2b) and DSA (Fig. 2c) were bound to the apical plasma membrane of the majority of superficial cells expressing UPs (Fig. 2a1, b1, c1) in normal human urothelium (Fig. 2a2, b2, c2). In Ta tumours, all three lectins were bound to UP-positive and -negative superficial cells (Fig. 2d–f2). In T1 tumours, only Jacalin was bound to superficial cells (Fig. 2g), while the binding of ACA (Fig. 2h) and DSA (Fig. 2i) was negative. Binding of ACA and DSA is present in normal and Ta, but not in T1 biopsy samples. Furthermore, four types of superficial urothelial cells were observed in the ex vivo samples: (1) UP-positive cells with lectin binding (Fig. 2a2, b2, c2, d2, f2, g2); (2) UP-negative cells without lectin binding (Fig. 2a2, d2, f2, h2, i2); (3) UP-positive cells without lectin binding (Fig. 2a2, b2, e2, f2, h2, i2); (4) UP-negative cells with lectin binding (Fig. 2b2, c2, d2, e2, f2, g2).

In vitro models showed that Jacalin, ACA and DSA bound evenly to the apical plasma membranes of all RT4 and T24 cells, but not so evenly to NPU cells (Fig. 3). Some NPU cells showed stronger Jacalin, ACA and DSA labelling than other NPU cells and than RT4 and T24 cells (Fig. 3a, d, g). These differences in lectin binding between cancer (RT4, T24) and normal (NPU) urothelial cells were confirmed by measuring the fluorescence intensities of the bound lectins (Fig. 3j). Jacalin and DSA showed significantly higher binding to NPU cells compared to both cancer cells (Fig. 3j). When the binding of Jacalin to RT4 and T24 cells was compared, the binding of Jacalin to T24 cells was significantly higher than to RT4 cells. ACA had higher binding to RT4 cells than to NPU and T24 cells (Fig. 3j). DSA had the highest binding to NPU, while the binding to RT4 and T24 was similar (Fig. 3j). As a control, the binding of Jacalin, ACA and DSA was tested after incubation with the corresponding inhibitory molecules galactose, mucin and chitin hydrolysate, respectively (Supplementary Fig. S1). FITC fluorescence intensity measurements showed a significant decrease in Jacalin, ACA and DSA binding after incubation with inhibitory molecule, indicating the specificity of lectin binding.

**Fig. 4:**
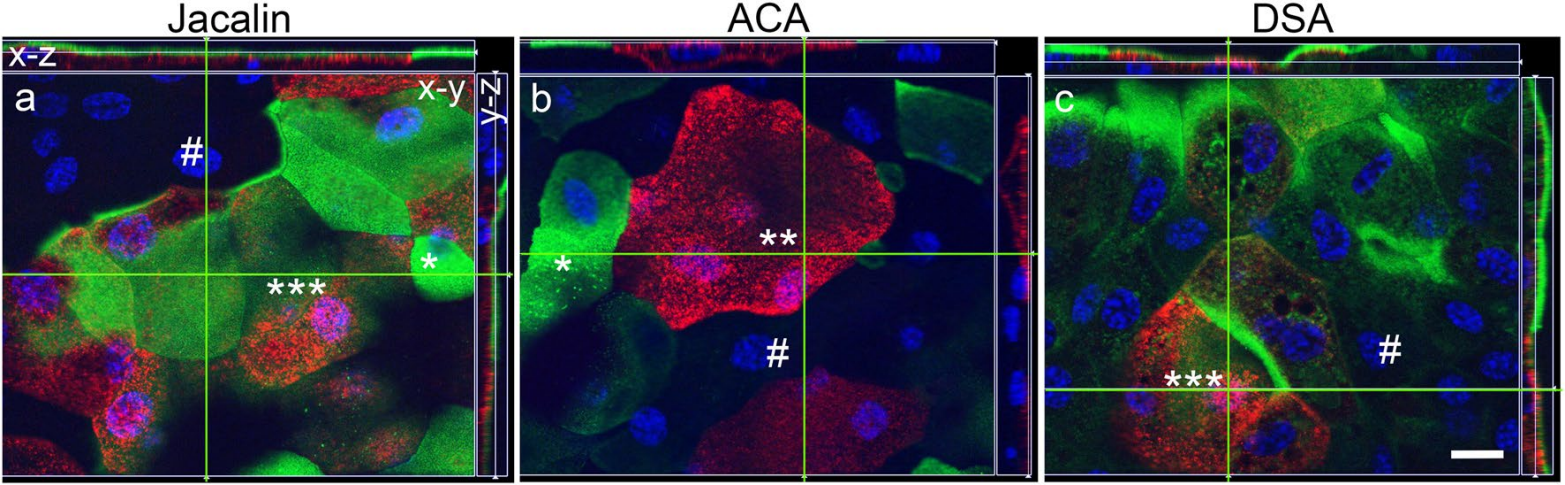
Correlation of FITC conjugated lectins Jacalin (**a**), ACA (**b**) and DSA (**c**) binding (green) and UP labelling (red) in NPU cells. UP-positive cells with lectins (**a**, **c**; three asterisks), UP-negative cells without lectin (**a**, **b**, **c**; hash), UP-positive cells without lectins (**b**; two asterisks), UP-negative cells with lectins (**a**, **b**; one asterisk). Shown are x–y, x–z and y–z views of optical sections. Scale bar: 10 µm

Correlation of lectins with UP labelling showed four types of NPU cells: (1) UP-positive cells with lectin binding; (2) UP-negative cells without lectin binding, (3) UP-positive cells without lectin binding; (4) UP-negative cells with lectin binding (Fig. 4a–c). Since RT4 and T24 cells are weakly positive or negative for UPs, respectively, the correlations of lectins with UPs were not performed.

Taken together, the results of ex vivo models showed that ACA and DSA selectively bind to normal urothelium and to low-grade Ta tumours. In vitro, Jacalin bound more strongly to high-grade T24 cancer cells than to low-grade RT4 cells, whereas ACA bound more strongly to low-grade RT4 cells than to high-grade T24 cells. However, the binding of Jacalin and DSA to NPU cells was even stronger than to RT4 and T24 cells and Jacalin had stronger affinity than DSA regardless of cell type. Four cell types were observed ex vivo and in vitro regarding UPs and lectin labelling, demonstrating the comparability of the in vitro models with the ex vivo biopsy samples. Not surprisingly, some normal urothelial cells were UP negative ex vivo (Fig. 2a1, b1, c1), although the samples were histopathologically classified as normal. Indeed, this phenomenon has been observed previously (Zupancic and Romih 2013).

### Endocytosis of lectins is found in cancer cells in vitro and tumours ex vivo, but not in normal urothelial cells

**Fig. 5:**
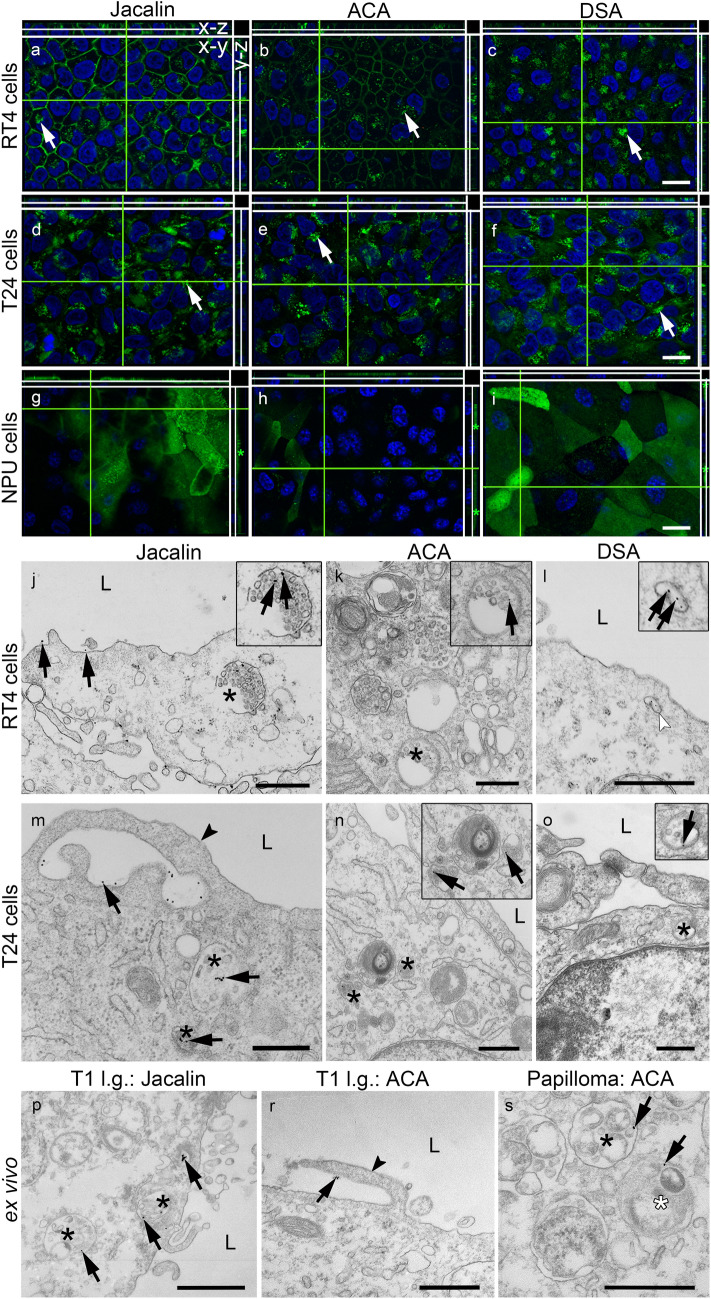
Endocytosis of FITC-conjugated lectins in urothelial cells in vitro and ex vivo. Jacalin (**a**, **d**), ACA (**b**, **e**) and DSA (**c**, **f**) were found in the cytoplasm of T24 and RT4 cells (white arrows). It is important to note that in NPU cells Jacalin (**g**), ACA (**h**) and DSA (**i**) are distributed only in the apical plasma membrane (y–z, green asterisk) and that the cytoplasm is without lectins. Shown are x–y, x–z and y–z views of optical sections. In the TEM images (**j**–**s**), the colloidal gold-conjugated lectins are indicated by black arrows. Jacalin is surrounded by membrane ruffles (m; arrowhead), which are an ultrastructural feature of macropinocytosis. After endocytosis, Jacalin (**j**, **m**; arrows), ACA (**k**, **n**; arrows) and DSA (**l**, **o**; arrows) are observed in MVBs (**j**, **k**, **m**, **n**, **o**; asterisk) and endosome-like vesicles (**l**; white arrowhead). The framed images are enlargements of the MVBs and vesicles with lectins. In the tumour biopsy samples ex vivo, the lectins (arrows) bind to the apical plasma membrane (**r**) and are associated with the plasma membrane ruffles (**r**; arrowhead). In the cytoplasm, lectins are gathered in multivesicular bodies (MVBs; **p**, **s**; black asterisk) and amphisome (**s**; white asterisk). L = lumen; l.g. = low grade. Scale bar: **a**–**i** 20 µm, **j**–**s** 500 nm

It is known that endocytosis is diminished in vitro in highly differentiated NPU cells (Kreft et al. 2009b; Tratnjek et al. 2017; Lojk et al. 2018) and undifferentiated in NPU cells (Resnik et al. 2021). To follow endocytosis of lectins in NPU cells, RT4 and T24 cells, live cells were incubated with FITC-conjugated lectins. In both RT4 and T24 cells, Jacalin (Fig. 5a, d), ACA (Fig. 5b, e) and DSA (Fig. 5c, f) were found as fluorescent dots in the cytoplasm, confirming endocytosis. Lectin binding to the plasma membrane of RT4 and T24 cells was present as well. In NPU cells, lectins were present at the apical plasma membrane but not in the cytoplasm (Fig. 5g, h, i).

Next, gold-conjugated lectins were used to detect ultrastructural features of endocytosis in RT4 and T24 cells and in tumour biopsies of bladder cancer ex vivo. Binding of lectins to the apical plasma membrane ruffles, which are typical features of macropinocytosis (Kerr and Teasdale 2009), were also detected in vitro and ex vivo via bound gold-conjugated lectins (Fig. 5m, r). Neither coated nor uncoated pits were observed to be filled with gold-conjugated lectins. We observed the lectins in macropinosomes, endosome-like structures and multivesicular bodies (MVBs) in RT4 and T24 cells (Fig. 5j–o), papilloma (Fig. 5s), Ta, l.g. (data not shown), T1 l.g. (Fig. 5p, r), T1 h.g. and T2 h.g. (data not shown) tumours. The ultrastructure and morphology of NPU, RT4 and T24 cells treated with gold-conjugated lectins corresponded to the ultrastructure of untreated cells (appearance of organelles, integrity of plasma membrane) (data not shown). In our previous study, the ultrastructure of NPU cells was also unchanged after WGA treatment (Kreft et al. 2009b). Furthermore, no necrotic or apoptotic features were observed. These observations confirm that the lectins were not cytotoxic.

Taken together, fluorescence microscopy confirmed endocytosis of lectins in cancer urothelial cells and not in normal cells. The ultrastructural features studied with TEM revealed macropinocytosis as endocytotic mechanism of lectin internalisation in in vitro and ex vivo models.

### Macropinocytosis is the main mechanism of lectin endocytosis in cancer urothelial cells

To date, several mechanisms of endocytosis into cancer cells have been described (Xiao et al. 2021; Hussein et al. 2021; Commisso et al. 2013). To test the mechanism of lectin endocytosis in cancer urothelial cells, we performed immunolabelling using antibodies against the major endocytotic markers caveolin, clathrin and flotillin, which showed no colocalisation of the individual endocytotic markers with the individual lectins (Supplementary Fig. S2-S4).

**Fig. 6:**
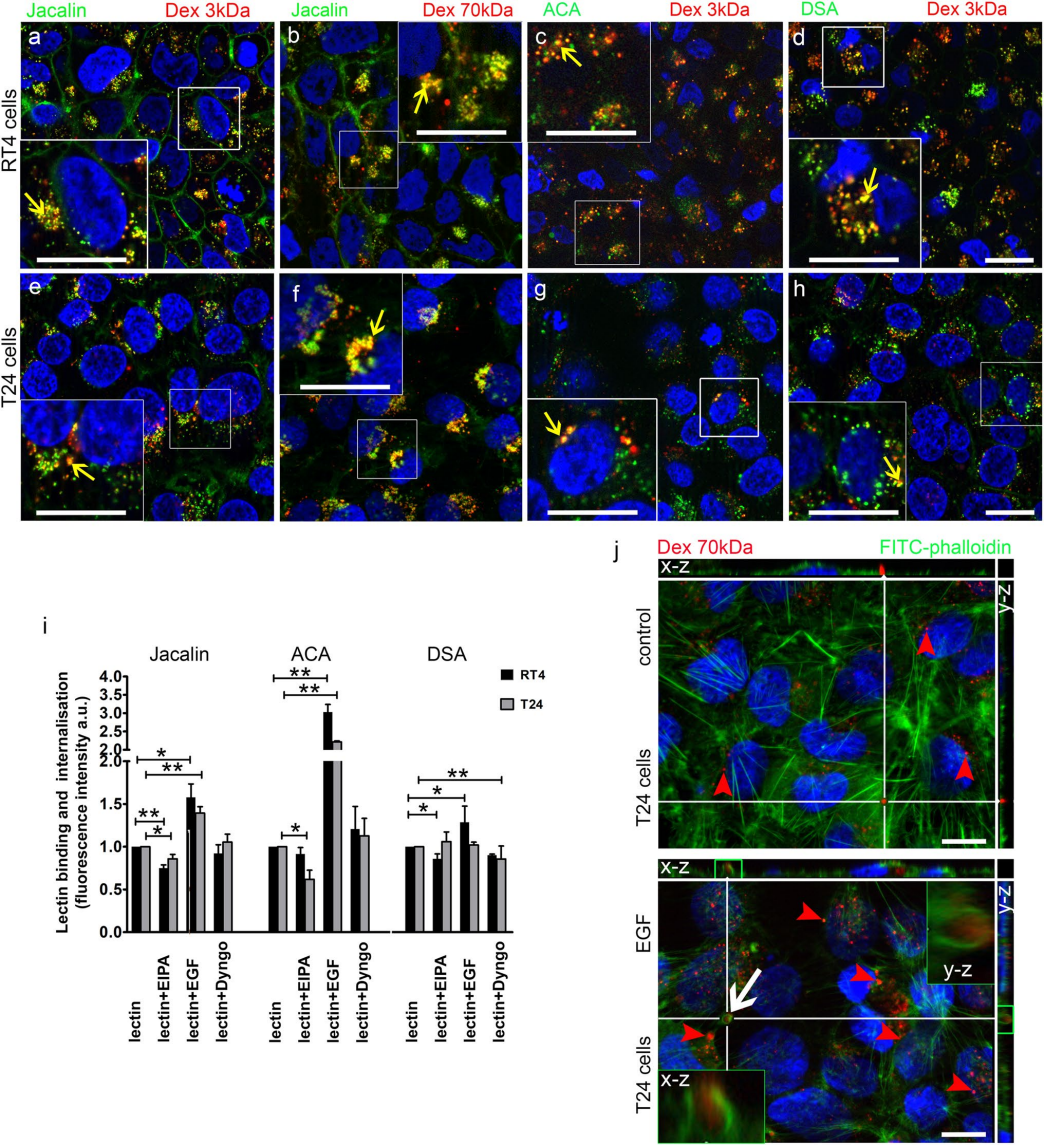
Macropinocytosis of the FITC-conjugated lectins Jacalin, ACA and DSA in cancer urothelial cells in vitro. (**a**, **e**, **c, d, g, h**) TRITC-conjugated dextran (3 kDa) (Dex 3 kDa, red), (**b**, **f**) TRITC-conjugated dextran (70 kDa) (Dex 70 kDa, red) and FITC-conjugated lectin (green) Jacalin (**a**, **b**, **e**, **f**), ACA (**c**, **g**) and DSA (**d**, **h**) colocalise in T24 and RT4 cells (yellow). Details of dextran and lectin colocalisation (yellow arrows) are shown enlarged in the white frames. (**i**) Quantification of lectin binding and internalisation in RT4 and T24 cells after treatment with EIPA (lectin + EIPA), EGF (lectin + EGF) and Dyngo (lectin + Dyngo) and without treatment (lectin). The intensities of the fluorescence were normalised to the endocytosis of the single lectin in cells without treatment (value 1). Shown are averages ± SE; *p < 0.05, **p < 0.001. (**j**) The distribution of TRITC-conjugated dextran (70 kDa) (red) and actin filaments (FITC-conjugated phalloidin; green) in T24 cells without EGF stimulation (control) and with EGF stimulation. In EGF stimulated cells, TRITC-conjugated dextran (70 kDa) (red) is surrounded by actin filaments (green) in the apical cytoplasm (white arrow and green frames in x–z and y–z views). EGF-stimulated cells have more macropinosomes than control (unstimulated) cells (red arrows). Scale bar: **a**–**h** 20 µm, **j** 10 µm

Confirmation that lectins are endocytosed via macropinocytosis in cancer urothelial cell TRITC-conjugated dextran with a low molecular weight of 3 kDa and a high molecular weight of 70 kDa was applied to cells together with FITC-conjugated lectins. Both 3-kDa and 70-kDa dextrans are capable of labelling macropinosomes (Chen et al. 2018). Furthermore, 70 kDa dextran enters cells predominantly via clathrin- and dynamin-independent and amiloride-sensitive macropinocytosis (Commisso et al. 2013; Li et al. 2015). In the cytoplasm of RT4 and T24 cancer cells, we observed colocalisation of dextran 3 kDa and Jacalin (Fig. 6a, e) to a similar extent as the colocalisation of dextran 70 kDa and Jacalin (Fig. 6b, f). Colocalization of TRITC-conjugated dextran (3 kDa) with ACA (Fig. 6c, g) and DSA (Fig. 6d, h) was also observed.

Additionally, we investigated the effects of inhibition of macropinocytosis on endocytosis of lectins. We used EIPA, the inhibitor of Na^+^/H^+^ exchange that specifically inhibits macropinosome formation, with no detectable effects on the other endocytic pathways studied (West et al. 1989; Koivusalo et al. 2010). EIPA caused a decrease in endocytosis of Jacalin in T24 and RT4 cells to 85.9% and 75%, respectively (Fig. 6i). The endocytosis of ACA was reduced to 61.8% in T24 cells after inhibition with EIPA. Endocytosis of DSA was reduced by EIPA to 85.8% in RT4 but not in T24 cells (Fig. 6i).

To stimulate macropinocytosis, we used EGF (Nakase et al. 2015). The results showed that endocytosis of Jacalin and ACA was increased in T24 and RT4 cells, while endocytosis of DSA was increased only in RT4 cells (Fig. 6i). The efficiency of endocytosis of Jacalin was increased to 139.2% in T24 cells and 157.7% in RT4 cells. Meanwhile, EGF stimulation increased the endocytosis of ACA to 221% in T24 cells and to 303% in RT4 cells (Fig. 6i). Fluorescence microscopy of Jacalin after EIPA and EGF treatment of RT4 cells also supported quantification (Supplementary Fig. S5). Jacalin fluorescence in the cytoplasm decreased after EIPA treatment and increased after EGF treatment compared with untreated RT4 cells (Supplementary Fig. S5). In EGF stimulated T24 cells, more dextran (70 kDa)-positive macropinosomes were observed compared with unstimulated (control) cells (Fig. 6j). In the presence of EGF, membrane ruffles at the plasma membrane engulfed dextran 70 kDa, indicating initiation of macropinocytosis (Fig. 6j). EGF was not used in NPU cells because they have minimal endocytosis (Lojk et al. 2018; Tratnjek et al. 2017; Kreft et al. 2009b).

To inhibit clathrin-mediated endocytosis, we used Dyngo (McCluskey et al. 2013). This inhibition caused a decrease to 85.7% in endocytosis of DSA in T24 cells and no significant decrease in endocytosis of DSA in RT4 cells (Fig. 6i). Furthermore, Dyngo did not alter the endocytosis of Jacalin and ACA in either RT4 or T24 cells (Fig. 6i). To exclude a clathrin-independent/dynamin dependent endocytosis (FEME; Fast Endophilin-Mediated Endocytosis), which is inhibited by EIPA (Boucrot et al. 2015), we conducted co-labelling of dynamin 2 and lectins. No colocalisation was observed among dynamin 2 and Jacalin, ACA and DSA (Supplementary Fig. S6) in RT4 nor T24 cells.

Taken together, these results, along with transmission electron microscopy analysis, suggest that macropinocytosis is the main mechanism for endocytosis of the Jacalin, ACA and DSA in RT4 and T24 cells.

## Discussion

We present here a way to selectively target cancer urothelial cells using lectins based on changes in the expression of sugar residues during urothelial cell dedifferentiation and cancer progression. Furthermore, we revealed that the main mechanism of lectin endocytosis in cancer urothelial cells is macropinocytosis.

Normal, highly differentiated urothelial cells in vivo and in vitro express UPs and form urothelial plaques at the apical plasma membrane (Hudoklin et al. 2011; Romih et al. 2002; Wu et al. 1994; Kreft and Robenek 2012). Here, we show that the expression of UPs is altered during transformation of cancer urothelial cells, as only some superficial urothelial cells of Ta, T1 and T2 tumours strongly express UPs, while the majority of superficial cells express UPs weakly or not at all. In addition, only single low-grade RT4 cells and none of the high-grade T24 cells express UPs (Fig. 1). Another feature of urothelial cancer transformation is the alteration of sugar residues exposed on the luminal surface (Ohyama 2008). However, it is not yet known whether lectins allow selective targeting in RT4 and T24 cells and in urothelial tumours. The later could be exploited for selective targeting of urothelial cancer cells by lectins.

Selective binding of the lectins ACA and DSA is observed ex vivo in cancer urothelial cells from biopsy samples of tumours compared to normal urothelium (Fig. 2). Meanwhile, higher binding of the lectins Jacalin and DSA is detected in vitro in normal urothelial cells NPU when compared to cancer urothelial cells RT4 and T24 (Fig. 3). Jacalin has the most widespread binding capacity ex vivo and shows binding to the apical plasma membrane of superficial urothelial cells in normal urothelium and all tumour biopsies. Binding of ACA and DSA is present in normal and Ta but not T1 biopsy samples (Fig. 2). These results are consistent with our previous study in the mouse bladder cancer model, where DSA binding decreased with cancer progression (Zupancic et al. 2014). Jacalin and DSA show similar binding capacity in vitro. Both lectins have significantly higher binding capacity in normal NPU cells than in high-grade T24 cells, and the lowest in low-grade RT4 cells, similar to the binding capacity of *Aleuria aurantia* (AAL) lectin (Ezeabikwa et al. 2021). However, Jacalin shows significantly stronger affinity than DSA regardless of the cell type. We hypothesise that this is because Jacalin is a polyspecific lectin that interacts with mannose, mannose-containing glycans, glucose, *N*-acetylneuraminic acid (NANA) and *N*-acetylmuraminic acid in addition to galactose (Bourne et al. 2002). Taken together, the patterns of Jacalin and DSA binding in vitro distinguish between urothelial normal and cancer cells. In addition, RT4 and T24 cells could be distinguished on the basis of higher Jacalin and ACA binding, as the binding capacity of Jacalin and ACA differs significantly between RT4 and T24 cells. This finding helps to identify a low- or high-grade tumour stage. However, this is not the case with biopsy samples. Here, the ability of Jacalin to distinguish among normal, Ta and T1 tumours is undetectable, presumably because of the differences between patients and within the tumour (Kang et al. 2020). Furthermore, T24 and RT4 cells differ from NPU cells in that they bind significantly less DSA. Our results of lectin binding ex vivo suggest that DSA could be used as a tool to distinguish DSA-negative T1 tumours from DSA-positive Ta tumours and normal urothelium. Whether these patterns of selective lectin binding have an even higher sensitivity could be investigated by using the extremely sensitive and accurate lectin microarray technology (Nishijima et al. 2012).

An important fact that could increase the selectivity of lectin targeting is the correlation between urothelial cell differentiation status and lectin binding. Similar to rat and mouse bladder cancer models (Zupancic et al. 2014), we show here in human urothelial tumours and urothelial cancer cell lines that there is no correlation between UP labelling and ACA, DSA and Jacalin binding. This is consistent with the fact that the glycan profile changes during cell differentiation (Park et al. 2015; McMorran et al. 2016; Higashi et al. 2014). The greatest biological significance in bladder cancer is the change in protein N-glycans, which include β1-6 branching of N-glycans due to overexpression of the enzyme GlcNAcT-V (GnT-V) and the addition of bisecting GlcNAc branches by GlcNAcT-III (GnT-III) glycosyltransferases (Takahashi et al. 2006). Alterations in the O-glycosylation pathways are also a common feature of malignant changes in the bladder, particularly the overexpression of simple mucin-type O-glycans and their sialylated counterparts, T-, sialyl-T- (ST), Tn- and sialyl-Tn- (STn) antigens. Presumably, the T and ST antigens that bind Jacalin and ACA are recognised, but these two lectins also bind to normal urothelium (Ferreira et al. 2013). The important difference is the binding capacity, which is higher in NPU cells than in RT4 and T24 cells for Jacalin and lower in NPU cells than in RT4 for ACA. Altered expression of the glycan end structures is also a common feature of bladder tumours. In particular, abnormally low or absent expression of ABO(H)-blood group termini is commonly found in high-grade and invasive bladder cancer (Bergman and Javadpour 1978), while low-grade bladder cancer cells express high levels of fucosylated Lewis X antigen (Ezeabikwa et al. 2021; Cordoncardo et al. 1988). Carbohydrate-terminal Lewis antigens are significantly under-expressed in normal urothelium compared to urothelial tumours (Cordoncardo et al. 1988; Ezeabikwa et al. 2021). Despite great progress in the glycobiology of bladder cancer, further experiments on the selective targeting of lectins and their endocytosis are still needed.

Selective targeting of lectins prompted us to investigate the mechanism of lectin endocytosis in RT4 and T24 urothelial cells and tumours. Lectins attach to the plasma membrane of urothelial cells and endocytose and localise in different endosomal compartments. For example, the lectin WGA is found in lysosomes and the trans‑Golgi network in human prostate cancer cells (Allen et al. 1989) or only in lysosomes in CaCo-2 cells (Wirth et al. 2002). TEM analysis of RT4, T24 cells and human biopsy samples shows that Jacalin, ACA and DSA are endocytosed and observed in macropinosomes, endosome-like vesicles, MVBs and amphisomes (Fig. 5). The morphology and ultrastructure of the RT4 and T24 cells were unchanged, confirming the non-toxicity of the lectin when these cells were incubated with the applied lectins. However, double labellings of lectins and clathrin, caveolin, flotillin and dynamin 2 show no colocalisation. We therefore assume that lectin endocytosis is not associated with clathrin-, caveolin-, and flotillin- and clathrin-independent/dynamin-dependent endocytosis (FEME) or at least to a small extent that is not detected by light microscopy. Recently, the glycolipid-lectin hypothesis has been presented, according to which glycolipids and lectins work together to promote the formation of tubular endocytic pits without the aid of cytosolic coats (Johannes et al. 2016; Renard and Boucrot 2021). However, the plant toxin ricin is endocytosed in HeLa cells via a macropinocytosis-like mechanism (Iversen et al. 2012). Moreover, there is ample evidence that cancer cells of various tumours use macropinocytosis mainly for nutrient uptake (Song et al. 2020; Stow et al. 2020). We therefore investigated the macropinocytotic pathway of lectins in RT4 and T24 cancer cells. We must emphasise that in vitro models such as RT4 and T24 cells do not directly correspond to the situation in vivo, but they are a good approximation and allow us to understand the mechanisms underlying the phenomena observed ex vivo.

Macropinocytosis has a dual effect on cancer cells (Song et al. 2020). On the one hand, cells expressing *RAS* genes (such as *K-RAS*, *H-RAS*) under the stress of nutrient deficiency can spontaneously generate constitutive macropinocytosis to promote cancer cell growth. On the other hand, abnormal expression of *RAS* genes and drug treatment can trigger a novel cell death associated with hyperactivated macropinocytosis, termed methuosis (Song et al. 2020). Because of this dual effect, there is immense potential for the development of anticancer therapies that target macropinocytosis in cancer cells. Furthermore, the molecular pathways and mechanisms of macropinocytosis contribute to the impressive adaptability of cancer cells. Different molecules such as EGFR, PTEN, V-ATPase, syndecan 1 and galectin-3 play distinct roles in the metabolic regulation of RAS-mediated macropinocytosis in cancer (Stow et al. 2020). Functional studies [i.e., using the macropinocytotic and clathrin-mediated endocytosis inhibitors EIPA and Dyngo, respectively, as well as the macropinocytotic stimulator EGF and co-localisation of dextran (3 and 70 kDa)] show that macropinocytosis is the main mechanism for endocytosis of Jacalin and ACA in RT4 and T24 cells (Fig. 6), while in T24 cells clathrin-mediated endocytosis predominates but is not significantly increased in T24 cells compared to macropinocytosis of DSA. This is to be expected, whereas T24 cells show increased macropinocytosis due to the endogenous oncogenic *RAS* mutation (Capon et al. 1983). On the other hand, these results are not confirmed by the double labelling of clathrin and DSA in T24 cells as no colocalisation can be detected here. According to Boucrot et al., EIPA also inhibits FEME, a clathrin-independent/dynamin-dependent endocytosis (Boucrot et al. 2015). Colocalisations between dynamin 2 and Jacalin, ACA and DSA in RT4 and T24 cells were negative, confirming that lectins are not endocytosed via FEME. The results of stimulation and inhibition of macropinocytosis are supported by transmission electron microscopy studies showing macropinocytotic structures (Fig. 5). The absence of lectin endocytosis in urothelial normal cells is not surprising and is consistent with our previous findings that urothelial normal cells have very low endocytotic activity (Kreft et al. 2009b; Lojk et al. 2018).

In conclusion, we suggest that anticancer drug-conjugated lectins are only endocytosed by cancer urothelial cells and could therefore represent a highly selective tool for the development of an effective intravesical targeted platform for the treatment of bladder cancer with minimal side effects on normal urothelial cells. Nevertheless, the potential toxicity of plant lectins should be considered as well as the fact that glycans are widely distributed in glycoproteins and glycolipids in body fluids, which affects selective targeting. Therefore, we suggest that intravesical application via a catheter is the best option to minimise the potential side effects of lectins. Another option is the use of lectins for glycan targeting in chimeric antigen receptor (CAR) T-cell therapy (Raglow et al. 2022). CAR T-cell therapy has already been used in the treatment of haematological malignancies, whereas it has not yet been successful in solid tumours. Since abnormally glycosylated glycoproteins and glycolipids are expressed on the surface of cancer cells, they are unique targets for CAR T-cell therapy. CAR T-system could use one or more specific lectins (e.g., ACA and DSA) for selective targeting of cancer cells, which would improve the efficacy of such therapy.

## Conclusion

Our study investigated the selective targeting and endocytosis of the lectins Jacalin, ACA and DSA in bladder cancer tumours from biopsies ex vivo and in vitro models of normal and cancer urothelial cells. The results show that discrimination between normal and cancer urothelial cells and between low- and high-grade cancer cells is possible by lectin binding. Furthermore, we confirm that macropinocytosis is the main mechanism of lectin endocytosis in cancer urothelial cells. Therefore, we propose that lectins should be used as targeting ligands for innovative drug delivery systems because of their enhanced uptake into cancer urothelial cells through macropinocytosis. In addition, lectins could also be used for glycan-binding chimeric antigen receptor (CAR) T-cell therapy in solid tumours that have different glycosylation patterns.

### Supplementary Information

Below is the link to the electronic supplementary material.

Supplementary file1 (DOCX 7847 KB)

## Data Availability

The datasets generated during and/or analysed during the current study are available from the first author and the corresponding author on reasonable request.
