# Supplementary material for: Selective targeting of lectins and their macropinocytosis in urothelial tumours: translation from in vitro to ex vivo
Source: Histochem Cell Biol. 2023 Aug 3;160(5):435–52. doi: 10.1007/s00418-023-02224-2 (PMC10624759; doi:10.1007/s00418-023-02224-2)
Supplement: Supplementary file 1 — Supplementary file1 (DOCX 7847 KB) [file 418_2023_2224_MOESM1_ESM.docx]

**Electronic Supplementary Material**

**Selective targeting of lectins and their macropinocytosis in urothelial tumours: translation from *in vitro* to *ex vivo***

**Histochemistry and Cell Biology**

Nataša Resnik ^1^, Tanja Višnjar ^2^, Tomaž Smrkolj ^3^, Mateja Erdani Kreft ^1^, Rok Romih ^1^, Daša Zupančič ^1,^*

^1^ Institute of Cell Biology, Faculty of Medicine, University of Ljubljana, Ljubljana, Slovenia

^2^ Clinical Institute of Genomic Medicine, University Medical Centre Ljubljana, Ljubljana, Slovenia

^3^ Department of Urology, University Medical Centre Ljubljana, Ljubljana, Slovenia

^4^ Department of Surgery, Faculty of Medicine, University of Ljubljana, Ljubljana, Slovenia

Corresponding author:

*D. Zupančič,

Institute of Cell Biology, Faculty of Medicine, University of Ljubljana, Vrazov trg 2, SI-1000 Ljubljana, Slovenia

E-mail: [dasa.zupancic@mf.uni-lj.si](mailto:dasa.zupancic@mf.uni-lj.si)

ORCID: 0000-0001-9492-5706

Tel.: +386 1 543 7686

**
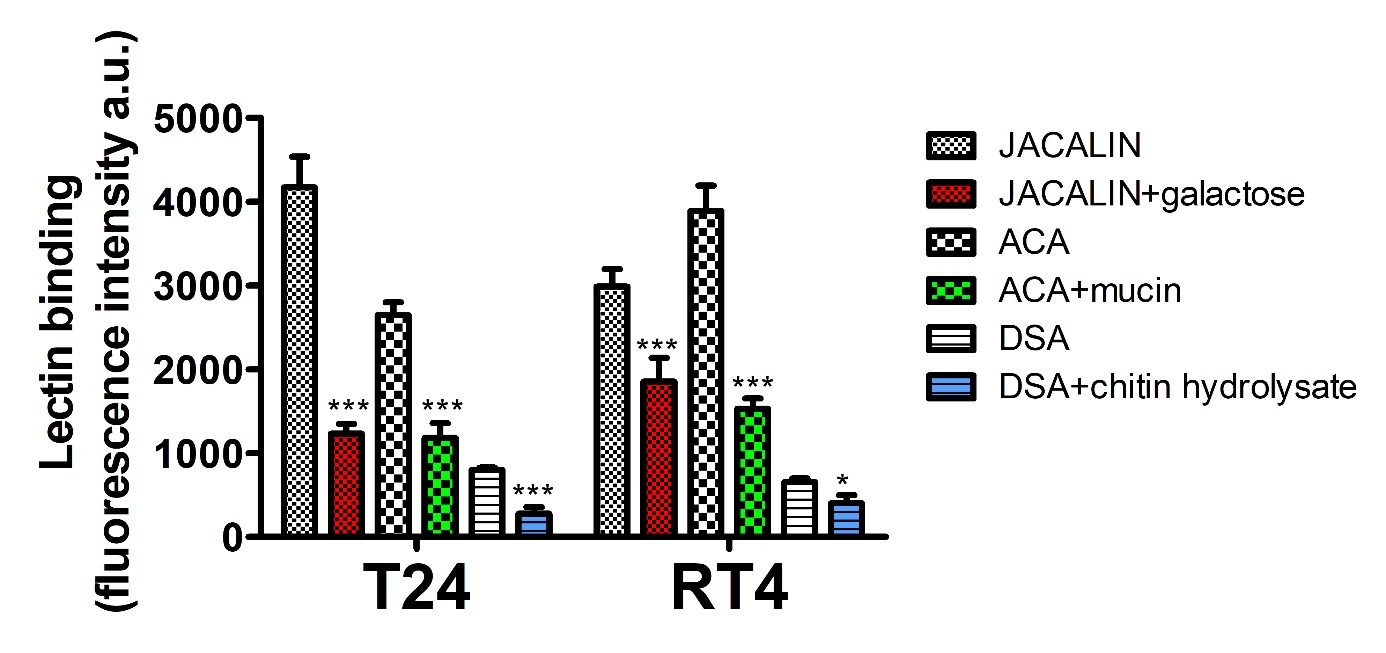
**

**Figure S1** Lectin binding to cancer urothelial cells *in vitro* after incubation with inhibitory molecules. Cells were incubated with lectins (Jacalin, ACA and DSA) or with lectins preincubated with inhibitory molecules (galactose, mucin and chitin hydrolysate). Lectin binding is presented as fluorescence intensities of FITC-conjugated lectins, averages between binding lectins alone or binding of lectins preincubated with inhibitory molecules are presented and *<0.05, **<0.001 denote significance between them.

**
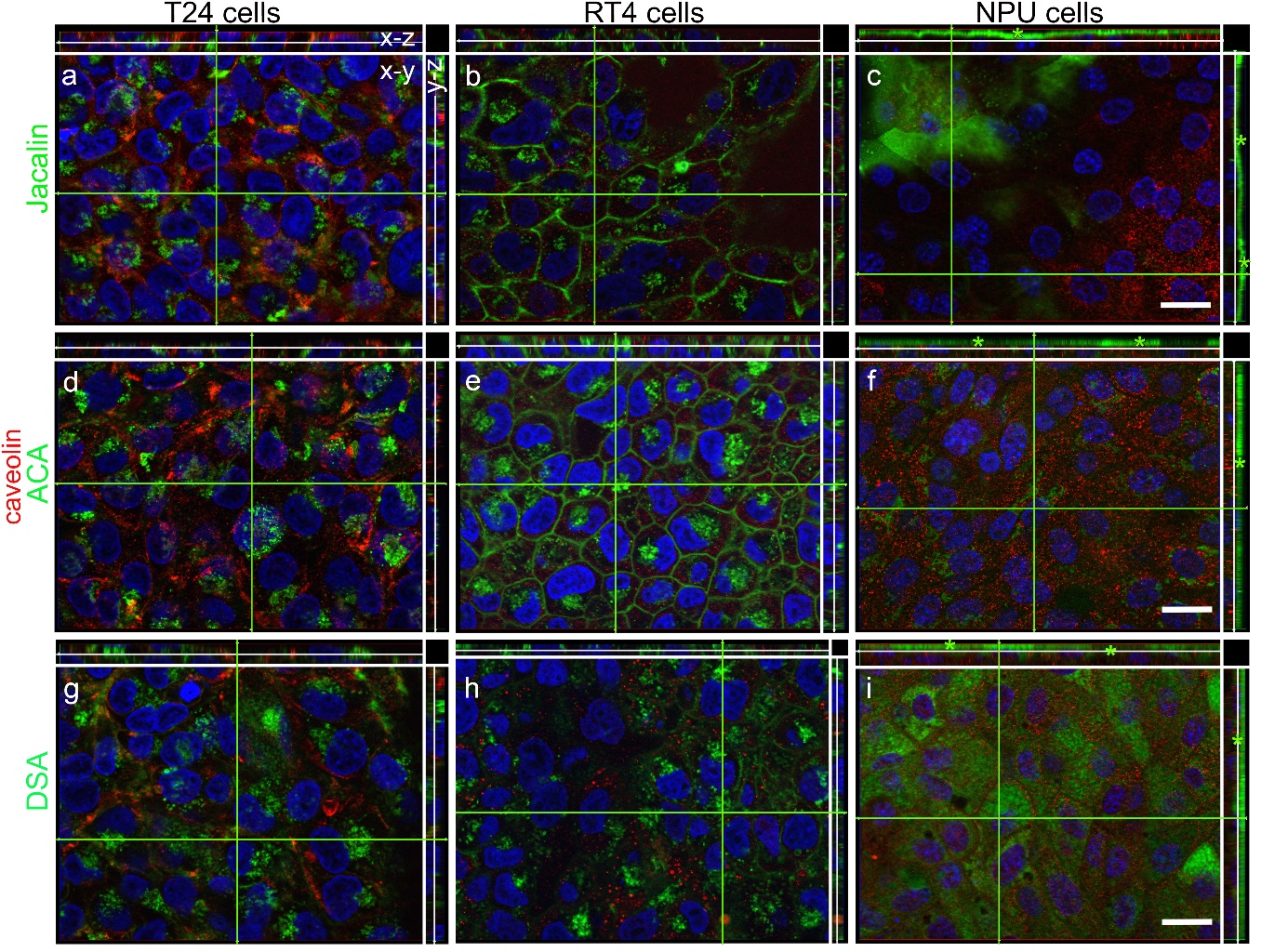
**

**Figure S2** Urothelial cancer cells RT4 and T24 do not colocalise lectins and the endocytotic marker caveolin. Lectins (green) are internalised in T24 and RT4 cells, but are not colocalised with caveolin (red), as shown in x-y, x-z and y-z views. In NPU cells, lectins bind to the apical plasma membrane (asterisks) and do not colocalise with caveolin. Scale bar: 20 µm.

**
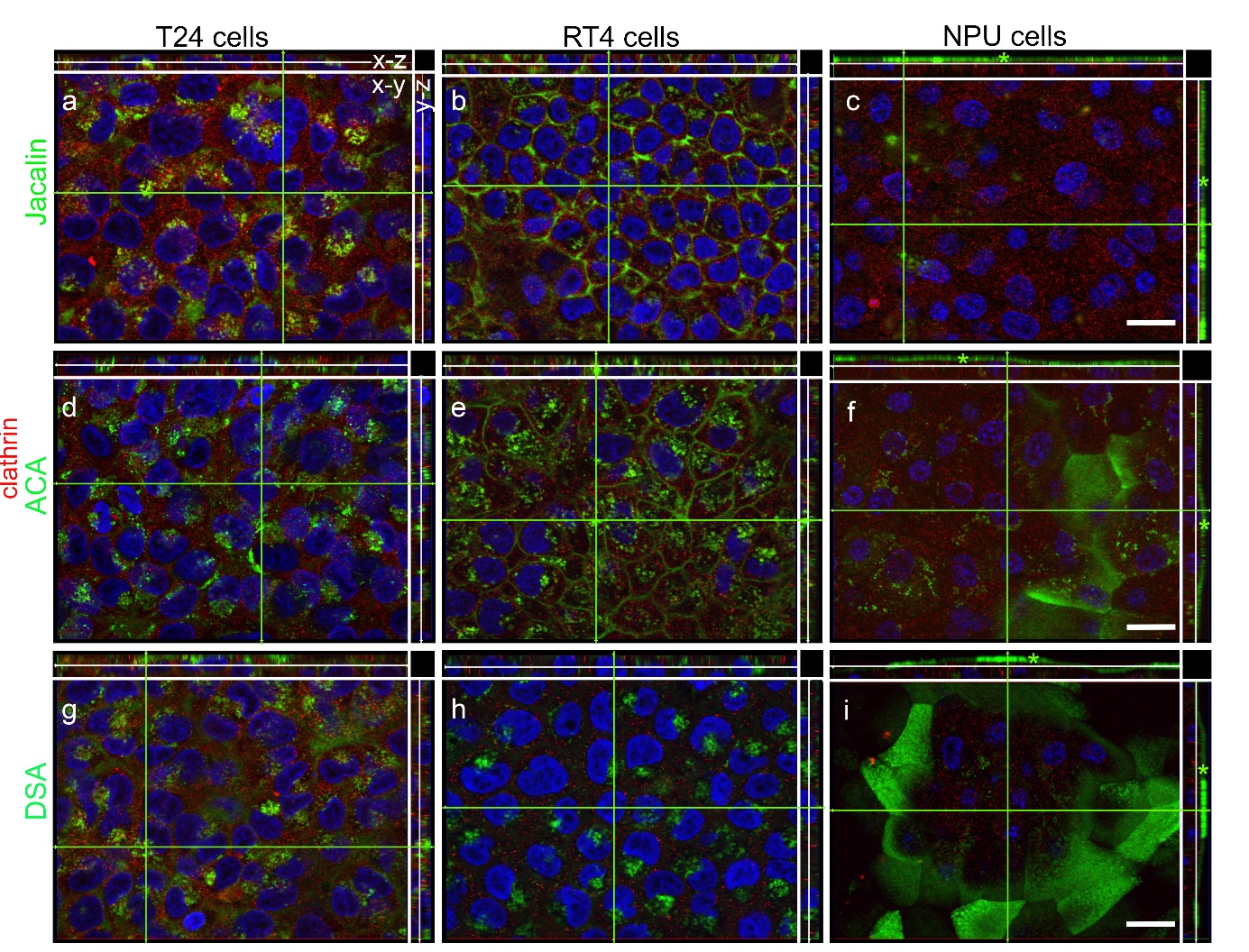
**

**Figure S3** Urothelial cancer cells RT4 and T24 do not colocalise lectins and the endocytotic marker clathrin. Lectins (green) are internalised in T24 and RT4 cell, but are not colocalised with clathrin (red), as shown in x-y, x-z and y-z views. In NPU cells, lectins bind to the apical plasma membrane (asterisks) and do not colocalise with clathrin. Scale bar: 20 µm.

**
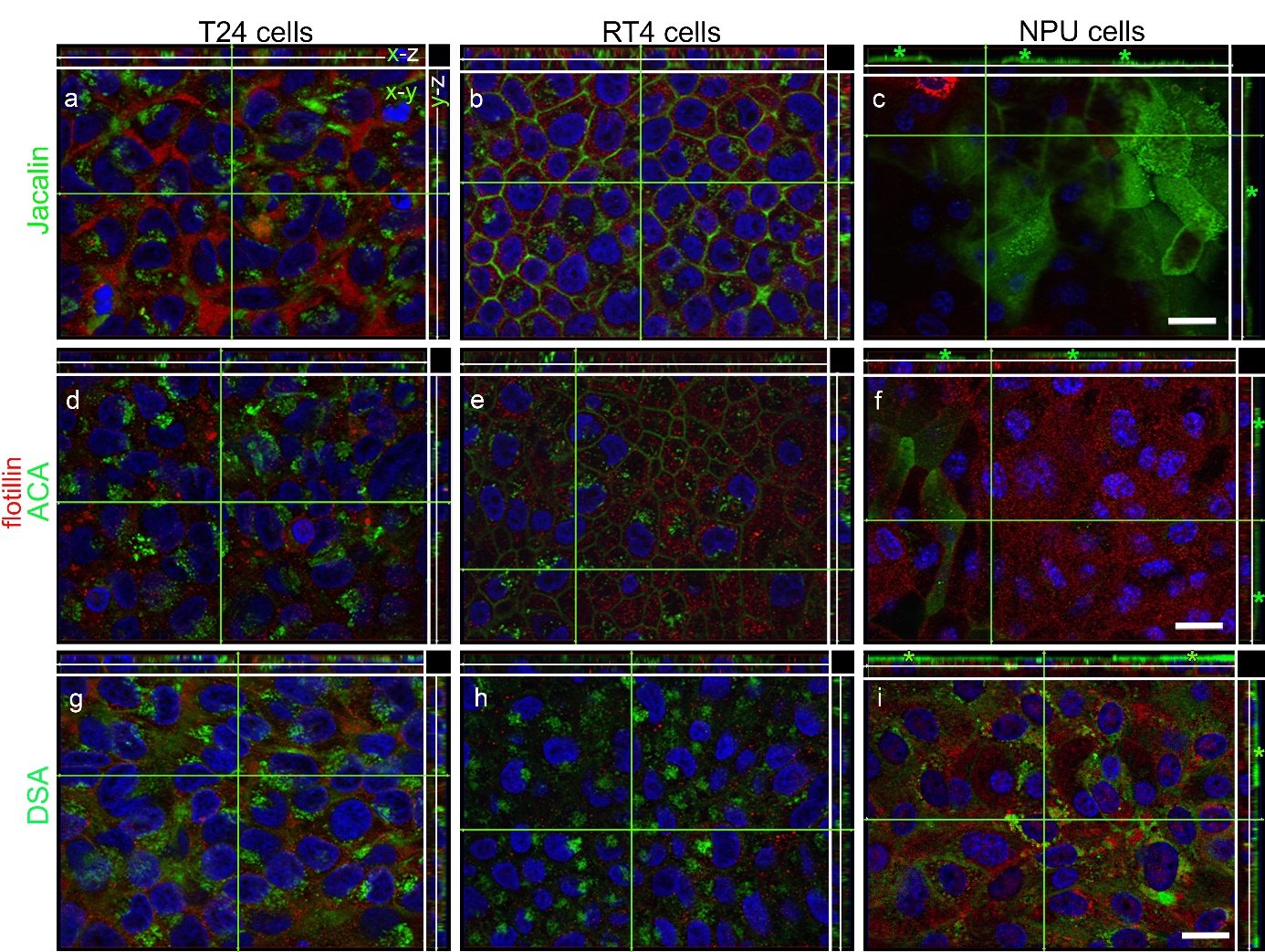
**

**Figure S4** Urothelial cancer cells RT4 and T24 do not colocalise lectins and the endocytotic marker flotillin. Lectins (green) are internalised in T24 and RT4 cells but are not colocalised with flotillin (red), as shown in x-y, x-z and y-z views. In NPU cells, lectins bind to the apical plasma membrane (asterisks) and do not colocalise with flotillin. Scale bar: 20 µm.

**
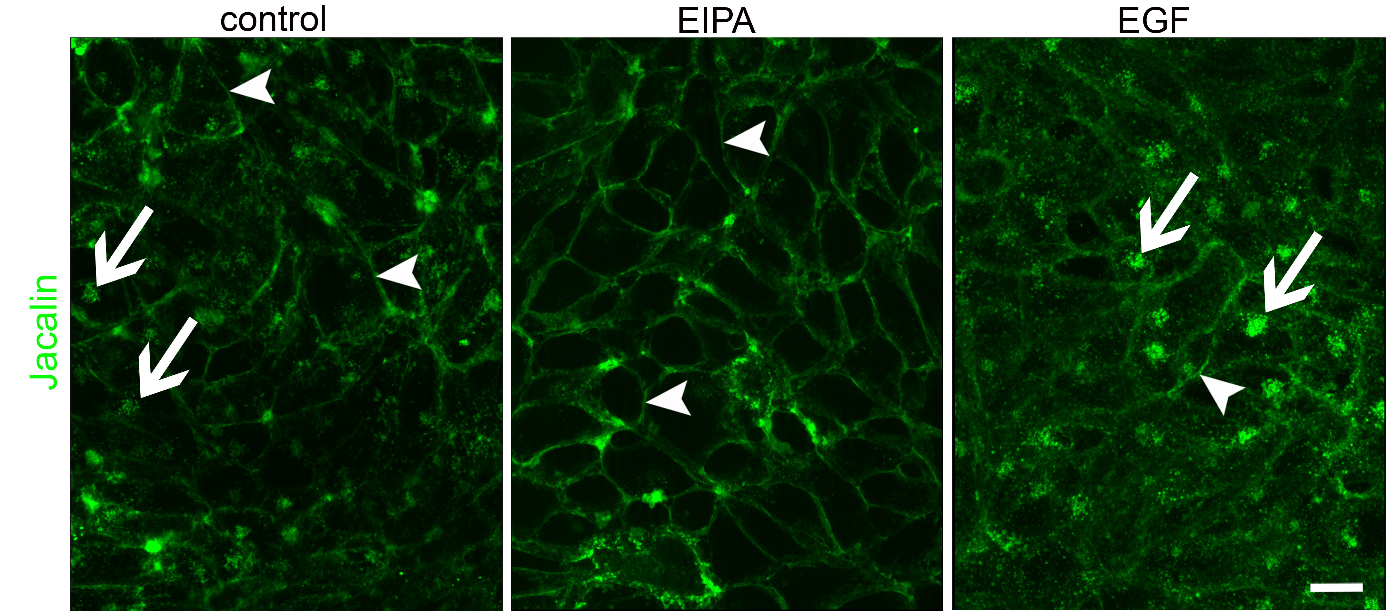
**

**Figure S5** FITC**-**Jacalin internalization in RT4 cells. Untreated RT4 cells (control) have Jacalin labelling on the plasma membrane (arrowheads) and in the cytoplasm (arrows). RT4 cells treated with EIPA have Jacalin labelling on the plasma membrane (arrowheads) and not in the cytoplasm. RT4 cells treated with EGF have intense Jacalin labelling in the cytoplasm (arrowheads). All images are maximum intensity projections (in z-direction) of a stack of images*.* Scale bar: 20 µm.

**
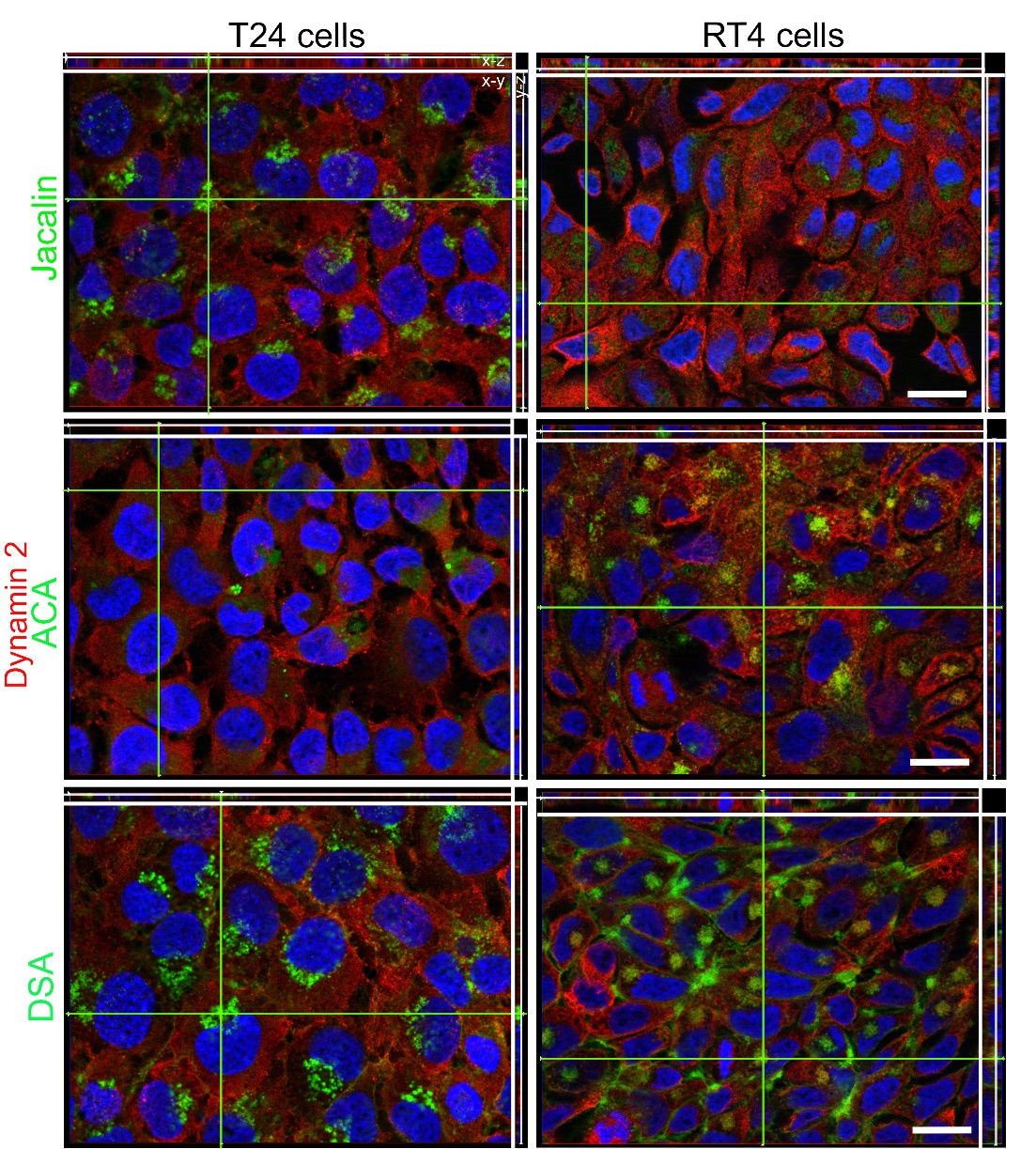
**

**Figure S6** Urothelial cancer cells do not colocalize lectins and dynamin 2. Lectins (green) are internalized into T24 and RT4 cells, but are not co-localized with dynamin 2 (red) as shown in x-y, x-z and y-z views. Scale bar: 20 µm.
